# Descemet’s Membrane Detachment during Phacocanaloplasty: Case Series and In-Depth Literature Review

**DOI:** 10.3390/jcm12175461

**Published:** 2023-08-23

**Authors:** Marta Orejudo de Rivas, Juana Martínez Morales, Elena Pardina Claver, Diana Pérez García, Itziar Pérez Navarro, Francisco J. Ascaso Puyuelo, Julia Aramburu Clavería, Juan Ibáñez Alperte

**Affiliations:** 1 Department of Ophthalmology, Lozano Blesa University Clinic Hospital, 50009 Zaragoza, Spainjascaso@gmail.com (F.J.A.P.); juanibanezalperte@msn.com (J.I.A.); 2 Aragon Health Research Institute (IIS Aragon), 50018 Zaragoza, Spain; 3 Department of Surgery, School of Medicine, University of Zaragoza, 50009 Zaragoza, Spain

**Keywords:** phacocanaloplasty, Descemet’s membrane detachment, open-angle glaucoma, Schlemm’s canal

## Abstract

This article presents three cases of Descemet’s membrane detachment (DMD) occurring during ‘ab externo’ phacocanaloplasty procedures in three patients with uncontrolled primary open-angle glaucoma (OAG) and discusses the management of this condition by reviewing the available literature. Following a successful 360° cannulation of Schlemm’s canal (SC), the microcatheter was withdrawn while an ophthalmic viscosurgical device (OVD) was injected into the canal. During passage through the inferonasal quadrant, a spontaneous separation of the posterior layer of the cornea was observed. Each case was managed differently after diagnosis, with the third case being drained intraoperatively based on experience gained from the previous cases. On the first postoperative day, slit-lamp biomicroscopy (BMC) revealed multiple DMDs in case one and a hyphema in the lower third of a deep anterior chamber. In the other two cases, a single DMD was observed. The second case developed hemorrhagic Descemet membrane detachment (HDMD), while the other two were non-hemorrhagic. In all three cases, anterior segment optical coherence tomography (AS-OCT) revealed the presence of retrocorneal hyperreflective membranes indicative of DMDs. These membranes were located in the periphery of the cornea and did not impact the visual axis. After evaluation, a small incision was made in the inferotemporal DMD of the first case. However, for the two remaining cases, a strategy of watchful waiting was deemed appropriate due to the location and size of the DMDs, as they did not affect the best-corrected visual acuity (BCVA). Over time, the patients demonstrated progressive improvement with a gradual reduction in the size of the DMDs.

## 1. Introduction

Canaloplasty is a well-established surgical technique for nonpenetrating glaucoma surgery that involves circumferential 360° catheterization and viscodilation of Schlemm’s canal (SC) to enhance the outflow of aqueous humor through the physiological collector system [1]. This article delves into the subject of iTrack Canaloplasty, also referred to as ‘ab externo’. The ab externo canaloplasty technique involves circumferential viscodilation of Schlemm’s canal, creating internal tension within the canal through the application of a tension suture. Nevertheless, recent studies have shown that complete 360° viscodilation without the need for sutures also yields favorable outcomes. These findings lend support to the significance of implementing the ab interno variation in this technique, utilizing an angle-based approach that is performed from within the eye [2]. While canaloplasty has shown a higher safety profile compared to conventional techniques, rare intraoperative and postoperative complications have been reported [3]. Konopinska et al. [4] recently reviewed the intraoperative complications of canaloplasty, identifying the most common complication as the inability to pass the microcatheter through SC, with an incidence ranging from 10 to 26%, and Descemet’s membrane detachment (DMD) occurring in 1.6 to 9.1% of patients. Alobeidan et al. [5] reported a 9.5% incidence of DMD in patients undergoing canaloplasty and phacocanaloplasty, noting that most cases of DMD occurred in combination with phacocanaloplasty. In this case report, we present three cases of DMD among a sample of 180 patients who underwent canaloplasty or phacocanaloplasty. These cases highlight the low incidence of this complication (1.67%) and provide additional evidence supporting the higher occurrence of DMD during phacocanaloplasty when compared to standard canaloplasty, particularly in patients with uncontrolled intraocular pressure (IOP) and open-angle glaucoma (OAG). This encompasses secondary glaucomas that present an open angle, such as pseudoexfoliative glaucoma. Additionally, we draw attention to the more frequent manifestation of DMD in the lower quadrants and discuss the management of this condition, given the absence of a consensus.

## 2. Case Series

### 2.1. Surgical Method

A standard ab externo SC surgery, known as canaloplasty ab externo, and phacoemulsification were performed under peribulbar anesthesia. Topical vasoconstrictive agents were applied to minimize bleeding. A corneal traction suture (7/0 vicryl) was placed at 12 o’clock to expose the upper area, followed by a limbal peritomy to expose the sclera. Minimal cautery was used for hemostasis, ensuring preservation of the episcleral collector channels. A partial thickness square flap measuring 4 × 4 mm was dissected according to the standard technique, followed by a deeper scleral flap (3 × 3 mm) created at approximately 95% depth, just above the choroidal tissue. At this stage, a standard phacoemulsification and a posterior chamber intraocular lens (AcrySof IQ IOL, Alcon^®^, Geneva, Switzerland) implantation were performed. The primary corneal incision was sutured using 10/0 nylon. Subsequently, the dissection of the inner scleral flap was continued until the SC was identified, crossing the scleral spur. The sides of the deep flap were dissected forward into the cornea creating a trabeculodescemetic window [6]. An ophthalmic viscosurgical device (OVD) with viscoadaptative properties (Healon GV; Johnson and Johnson, New Brunswick, NJ, USA) was injected through the SC opening on both sides. The canal was probed 360° using a microcatheter (iTrack^®^; Nova Eye Medical, Adelaide, Australia), with the surgeon tracking the illuminated tip of the catheter, aided by dimmed microscope lights. During the retraction of the catheter, OVD was injected at every 2 clock hours to dilate the canal, as directed by a well-trained assistant surgeon. The injected volume was controlled by a quarter turn of the syringe provided by the company, equivalent to 0.5 μL. While retracting the catheter, at the 6 o’clock position, a DMD was observed, accompanied by significant blood leakage mixed with viscoelastic material in the lower quadrants (inferonasal and inferotemporal quadrants in case 1, inferotemporal quadrant in cases 2 and 3), resembling a possible micro-rupture of the trabecular meshwork. In the third case, intraoperative management involved draining the DMD with a 30-gauge needle and intracameral air injection. Following that, the deeper scleral flap was excised, while the superficial flap was positioned and sutured tightly with 10/0 nylon to ensure closure of the trabeculodescemetic window. The primary objective was to prevent leakage through the filtering bleb instead of facilitating the exit of fluid through the collector channels. The anterior chamber was reformed with a balanced salt solution, and an air bubble was left to occupy the entire anterior chamber (AC). The conjunctiva was closed using a non-absorbable suture (8/0 silk).

All three procedures were performed using the same method and by the same surgeons, with no noticeable variations observed.

All the data regarding intraocular pressure provided below have been measured using Goldmann applanation tonometry, while the best-corrected visual acuity (BCVA) assessments were conducted using the Snellen chart.

### 2.2. Case 1

A 68-year-old woman, who was being monitored by the glaucoma department due to medically uncontrolled primary OAG, was selected as a candidate for a non-penetrating glaucoma canaloplasty surgery. Prior to the procedure, her BCVA was 1.0 and 0.3 in the right and left eyes (RE and LE), respectively. Ophthalmic refraction showed a slightly hyperopic prescription (+1.50 −0.50 90° in the RE and +0.25 +0.25 180° in the LE). Slit-lamp biomicroscopy (BMC) examination with a Topcon Slit Lamp Imaging System (SL-D701, Topcon Healthcare^®^, Oakland, NJ, USA) revealed the presence of a corticonuclear cataract in the LE. The IOP measured using Goldmann applanation tonometry while the patient was on maximum topical antiglaucoma medications ranged from 26 to 28 mmHg in the LE. The RE only required a single topical antihypertensive drop, and the IOP was well-controlled. Given the relatively slow progression of the disease and the IOP being below 30 mmHg, canaloplasty was selected as the treatment option [7]. 

**Figure 1:**
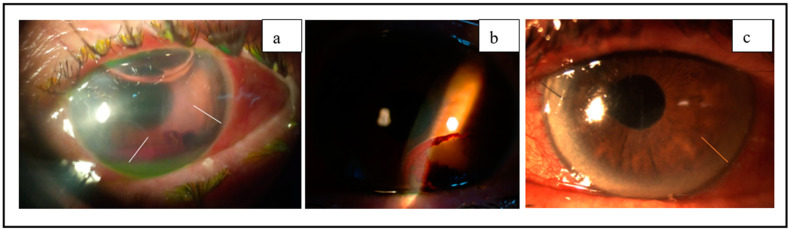
The BMC examination revealed two DMDs in the inferior quadrants, depicted by the white lines, along with a hyphema in the anterior chamber. Additionally, an air bubble was observed in the superior part of the anterior chamber (**a**). The second image (**b**) displays a hemorrhagic Descemet membrane detachment in the inferior temporal quadrant. Finally, the third image (**c**) shows a non-HDMD in the inferior temporal quadrant, indicated by the orange line.

On the first day after the surgery, slit-lamp BMC showed two transparent DMDs in the inferonasal and inferotemporal quadrants, measuring approximately 4 × 4 mm and 4 × 5 mm in diameter, respectively. Additionally, a 3 mm hyphema was observed in the lower region of a well-defined AC, which did not affect the visual axis (Figure 1a). The transparent appearance of the DMDs confirmed the presence of viscoelastic material and aqueous humor within them, and they did not contain hematic material.

### 2.3. Case 2

A 55-year-old woman diagnosed with uncontrolled primary OAG was chosen as a candidate for a combined phacocanaloplasty procedure. The patient was receiving treatment with topical prostaglandin analogs, and the Goldmann applanation tonometry measured the IOP at 24 mmHg. In terms of her ophthalmological history, the RE had previously undergone a successful phacoemulsification combined with non-penetrating deep sclerectomy (NPDS) surgery in 2020. Prior to the surgery, the BCVA was 1.0 in the RE and 0.3 in the LE. Slit-lamp BMC examination revealed the presence of a nuclear cataract and mild conjunctival hyperemia.

After a 24-h postoperative assessment, BMC examination showed corneal stromal edema and hemorrhagic Descemet membrane detachment (HDMD) in the lower temporal sector, measuring approximately 4 × 4 mm in diameter. The HDMD did not affect the visual axis, and an air bubble was observed in the upper AC. The IOP measured at this time was 17 mmHg.

### 2.4. Case 3

A 79-year-old woman was diagnosed with open-angle pseudoexfoliative glaucoma and had a history of trabeculectomy in her RE. Due to progressive glaucoma in her LE with an IOP of 27 mmHg, despite treatment with prostaglandin analogs and topical beta-blockers, it was decided to perform phacocanaloplasty. Slit-lamp BMC examination revealed the presence of a corticonuclear cataract. The BCVA prior to the surgery was 0.6.

On the first day after the surgery, a significant corneal edema was observed, which hindered the clear visualization of the inferior temporal DMD that was noted during the intraoperative period. The IOP measured at this time was 12 mmHg.

In all three cases, a treatment regimen consisting of topical antibiotics and corticosteroids was initiated, along with anti-edematous medication.

**Figure 2:**
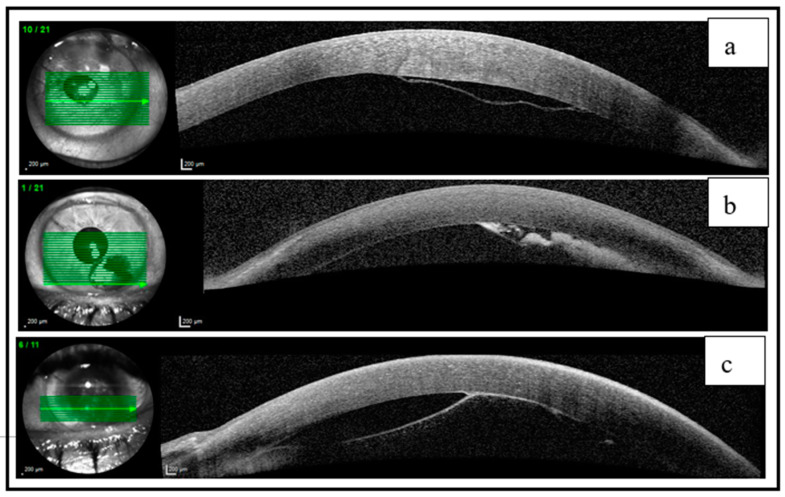
On the first postoperative day, AS-OCT of the LE revealed the presence of distinct DMDs in the inferior quadrants. The green lines represent horizontal cross-sections of the cornea, indicating the precise locations where the OCT scan has been performed.

Anterior segment optical coherence tomography (AS-OCT) imaging using the Spectralis System from Heidelberg Engineering^®^, Heidelberg, Germany, displayed the presence of a retrocorneal hyperreflective membrane in all three cases. This membrane formed a pseudo-chamber with sinuous edges and contained a hypointense material corresponding to a DMD (Figure 2a,c). Additionally, in case 2, AS-OCT showed a hyperintense material corresponding to an HDMD (Figure 2b). 

The peripheral locations of the DMDs observed did not extend to the visual axis. Following the diagnosis, the DMD in the temporal position of case 1, which was larger and closer to the visual axis, was drained using a precise cornea blade. However, for the remaining cases, an initial period of expectant management was deemed suitable since the location and size of the DMDs did not have an impact on BCVA. In case 2, multiple follow-up visits were scheduled to closely monitor the hematic collection.

## 3. Results

**Figure 3:**
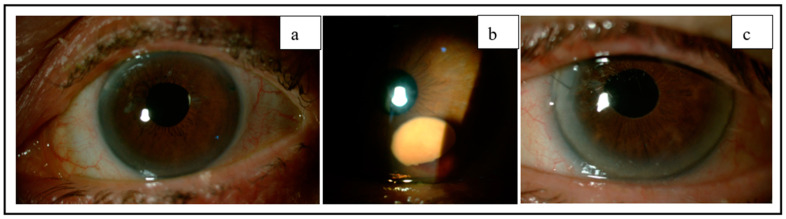
Three months after the surgery, BMC examination shows the progression of the DMDs in the lower corneal segment.

**Figure 4:**
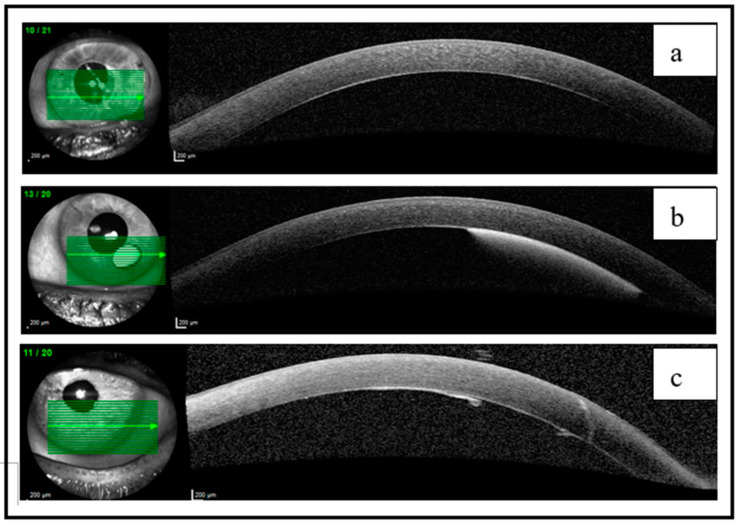
Three months post-surgery, the SA-OCT of the LE reveals different findings: in (**a**), there is a complete reapplication of the Descemet membrane after three months of observation; (**b**) shows a remaining intracorneal stain that does not have an impact on vision; in (**c**), a minor disruption of the DM is observed, but there is no corneal edema present.

The patients demonstrated positive progress during the 3-month follow-up period, with gradual improvement. In case 1, where the DMD was drained immediately after surgery, a faster resolution was observed. Follow-up examinations with BMC and SA-OCT indicated a gradual reduction in the detachment, eventually leading to its complete reapplication, and the BCVA after 3 months was 0.9 (Figure 3a and Figure 4a). In case 2, the HDMD underwent oxidation, resulting in a residual pre-Descemetic intracorneal orange spot. However, this spot did not have any impact on vision, and the BCVA after 3 months was 1 (Figure 3b and Figure 4b). In case 3, a minor localized disruption of Descemet’s membrane was visible, without causing edema or affecting vision, and the BCVA after 3 months was 0.9 (Figure 3c and Figure 4c). Six months post-surgery, the patients achieved a BCVA of 0.9–1, and their IOPs ranged between 12 and 15 mmHg. There was no evidence of structural or functional progression in their glaucoma condition (Table 1).

## 4. Discussion

This research paper focuses on three cases of DMD that occurred in our center following phacocanaloplasty procedures. It is important to emphasize that this complication was observed exclusively in cases where combined surgery was performed, and it did not arise during standard canaloplasty procedures. It is worth highlighting that our center possesses a wealth of expertise and experience in performing canaloplasty and phacocanaloplasty procedures, having successfully completed a total of 180 interventions of this nature. This extensive experience contributes to our understanding and management of complications such as the DMDs observed in these cases.

Canaloplasty is a minimally invasive surgery for glaucoma that has proven effective in treating mild-to-moderate OAG and has demonstrated an excellent safety record [7]. Numerous studies have compared outcomes and complication rates between various glaucoma treatment techniques. In comparison to trabeculectomy, canaloplasty has shown similar postoperative success rates, lower complication rates, and higher patient satisfaction [3,8,9,10,11]. One of the major advantages of canaloplasty is its minimal complications and their limited clinical significance, particularly when compared to the presence of a filtering bleb. The secure closure of the superficial scleral flap eliminates patient discomfort, positive Seidel’s test indicating wound leakage, and intraocular infections [7]. Hyphema, characterized by the reflux of blood from the SC towards the AC, is a common occurrence (with reported incidences ranging from 6.1 to 70%) resulting from the difference in intraocular and intravenous pressure [12,13,14]. This phenomenon is considered an early sign of the procedure’s success, as it indicates the patency of natural drainage pathways [12]. DMD is a rare complication following nonpenetrating glaucoma surgery, including canaloplasty. Following standard phacoemulsification, recent reports indicate an occurrence of DMD ranging from 0.044% to 0.52% [15]. The reported incidence of DMD after canaloplasty varies from 1.6 to 9.1% [5,6,12,16,17,18,19], which is comparable to the findings of our study. In our center, we have conducted a series of 180 interventions over the past few years. The sample was carefully chosen to ensure comparability, and consisted of a population group that ensured comparability, with an equal distribution of men and women and similar average age. All surgeries were performed by the same surgical team, using a standardized technique and without any reported errors. The learning curve and guidance from the principal surgeon to the assistant surgeon are crucial, as a rapid withdrawal of the microcatheter or excessive pressure during sodium hyaluronate injection in the SC (more than one click or 1/8 turn every 2–3 clock hours) can lead to this complication [5]. Of the 180 surgeries, we observed DMD in three cases (1.67%). As mentioned earlier, the detachments in our study occurred after the withdrawal catheter and viscodilation procedure.

During canaloplasty, the occurrence of DMD is more frequent in one of the inferior quadrants [4], which aligns with our observations. This increased frequency in the inferior region is believed to be attributed to the higher pressure induced in that specific area. There are a few explanations for the different causes of this complication. The most widely supported hypothesis suggests that during the withdrawal of the microcatheter, the viscoelastic material injected may accumulate to a critical mass in the inferior quadrants of the SC, where it cannot exit easily through the ostia of the canal as it does in the upper quadrants. Furthermore, this situation may be exacerbated by a recoil effect in the viscoelastic material following the actuation of the device. The resistance within the canal may exceed the durability of the termination of DM at Schwalbe’s line, leading to a prolapse of the viscoelastic material and triggering blood reflux between the corneal stroma and Descemet’s membrane (DM) [4,5,20]. This intraluminal overpressure can occur due to obstructions or anatomic factors that make the cornea susceptible to weak adherence [13]. Additionally, bilateral DMD has been documented [21].

In our series of cases, all instances of DMDs occurred in the lower quadrants of the cornea and did not affect the visual axis. This percentage aligns with findings from other published articles [1,4,5].

**Figure 5:**
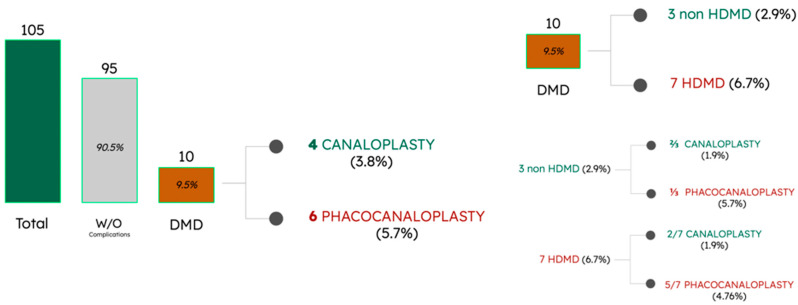
Incidence of DMD described by Alobeidan et al. [5], classified based on the type of surgery performed and the specific type of DMD.

**Figure 6:**
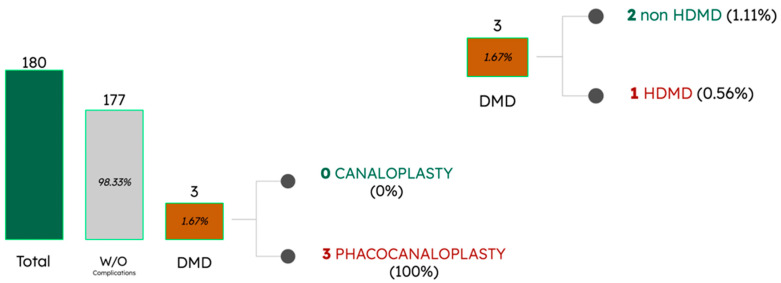
Incidence of DMD in our group, classified based on the type of surgery performed and the specific type of DMD.

Furthermore, based on our experience and from the existing literature [5], we have observed that this complication is more commonly seen in cases where combined surgery, specifically phacoemulsification combined with canaloplasty, is performed. This could be attributed to greater intraoperative hypotonia during these combined procedures. Alobeidan et al. [5] reported a 9.5% incidence of DMD in patients undergoing canaloplasty and phacocanaloplasty. Notably, they found that six out of ten cases occurred in combination with phacocanaloplasty and most cases of HDMD were observed in this group (Figure 5). It is worth mentioning that all our cases of DMD were encountered in the context of combined surgery (Figure 6).

These figures aim to illustrate the low frequency of the complication in various series, emphasizing the key significance of the type of surgery performed and the subtype of DMD.

DMDs can be classified into two primary categories: hemorrhagic and non-hemorrhagic. This distinction is crucial in managing this complication. As previously mentioned, DMDs commonly occur in the lower areas of the cornea after canaloplasty. The lower nasal region is particularly rich in collector channels, which connect episcleral veins to the SC [22]. This anatomical arrangement can result in the reflux of blood within the space formed by the DMD. 

Another potential factor that can trigger blood reflux from the collector channels and contribute to HDMD is the aforementioned hypotonia during phacoemulsification in the same procedure [5,21]. Additional factors that can contribute to HDMD include the use of anticoagulant medications, anatomical changes in the SC such as changes in diameter and location [23], as well as preexisting stromal adherence to the Descemet membrane [24]. Collectively, these factors influence the development and severity of HDMD.

The management of the DMD depends primarily on its size, location, and content. Kumar et al. [25] proposed the HELP algorithm, which utilizes the height, extent, and relationship to the pupil based on AS-OCT for managing post-phacoemulsification DMD [15]. Non-conservative management is typically required for large detachments, especially those involving the visual axis or containing hemorrhagic DMD, and it is advisable to evaluate the risks/benefits and potentially withdraw anticoagulant medication. Various techniques have been described in the literature, such as neodymium: yttrium–aluminum–garnet (Nd: YAG) laser Descemet membranotomy, which is used to treat pre-Descemet hemorrhage [1,26]. This procedure helps prevent corneal blood staining and preserves corneal endothelium. It should be performed when the cornea appears free from opacities [27]. A recent novel treatment for severe cases of HDMD was described by Hamid et al. [28]. They suggested the use of tissue plasminogen activator (TPA) to quickly resolve the HDMD.

When surgical intervention is required [25], descemetopexy has emerged as the gold standard approach for managing DMD. This surgical technique involves filling the AC with an isoexpansile gas, such as perfluoropropane (C3F8) or sulfur hexafluoride (SF6), using a 26- or 30-gauge needle [29]. It is recommended to perform the paracentesis away from the DMD where the DM is still attached. The procedure begins with continuous drainage of aqueous fluid, followed by the injection of the gas [20]. Initially, descemetopexy was performed with air; however, its rapid absorption led to the adoption of isoexpansile gases [30]. 

To avoid postoperative pupillary block, the size of the gas bubble should be reduced to two-thirds of the AC. If a complete gas-filled chamber is left, it is advisable to perform an inferior peripheral iridectomy. After the procedure, the patient must maintain a supine position until the gas bubble is reabsorbed and the DM is reattached. 

Considering that this procedure might lead to a raised IOP and pupillary block glaucoma in 11.6% of patients [29,30], the use of cycloplegic drops and preoperative laser peripheral iridotomy to prevent this complication could be a prudent approach, especially in glaucomatous patients [15,31].

Conservative therapy for managing the condition involves the use of topical steroids to control inflammation and reduce the fibrosis. Additionally, hyperosmotic agents can be employed to clear corneal edema and improve VA [15]. 

In our case, due to its broader extension and closer proximity to the central cornea, we chose the less invasive approach for pre-Descemet drainage using a blade incision on the inferotemporal region of the DMD [32,33]. The interface was then rinsed with a balanced saline solution to remove any remaining OVD. 

When the type and duration of the OVD used are known, the material gradually disappears, allowing the Descemet’s membrane to coapt without further manipulation. 

Several authors have recommended expectant management for small detachments with regular edges located in peripheral regions and containing visco-aqueous content, as these cases have been observed to reattach spontaneously [34]. 

## 5. Conclusions

In conclusion, Descemet’s membrane detachment (DMD) represents a relatively infrequent but significant complication, particularly in the context of glaucoma and cataract surgeries. Despite the advancements in surgical techniques and understanding, the precise etiology of this complication remains elusive. While suspicions exist regarding the potential role of micro-ruptures within Schlemm’s canal during viscodilation, further research is required to establish a definitive cause.

To address the challenge of DMD prevention, meticulous attention and caution during viscodilation procedures are paramount. Implementing strategies to minimize the risk of micro-ruptures, such as precise surgical manipulation and controlled pressure application, could potentially contribute to reducing the incidence of DMD. 

Furthermore, it is imperative to recognize the considerable advantages that combined surgeries offer in terms of convenience and lowered healthcare expenses. Nevertheless, it is crucial to maintain awareness regarding the potential susceptibility to DMD resulting from postoperative hypotony.

In cases where DMD extends and impacts the visual axis, prompt intervention through external drainage techniques becomes imperative. By ensuring the preservation of best-corrected visual acuity (BCVA) and preventing the progression of visual impairment, these interventions play a critical role in maintaining patients’ ocular health and overall quality of life.

For smaller cases of DMD that do not encroach upon the visual axis, an approach of vigilant observation and conservative management is often warranted. Regular monitoring and periodic assessments are essential in order to promptly detect any changes that might necessitate intervention.

Continued research efforts are necessary to deepen our understanding of the mechanisms underlying DMD and to develop more precise preventive measures and effective management strategies. Collaborative endeavors between ophthalmologists and researchers are pivotal in advancing our knowledge and refining clinical practices to further enhance patient outcomes and minimize the impact of Descemet’s membrane detachment in ophthalmic surgeries.

## Figures and Tables

**Table 1:** Baseline clinical features and visual outcome of DMDs.

Subject	Baseline BCVA	Surgery	Type of Dmd	Location of DMD	BCVA after DMD	Intervention	BCVA after 3 Months	Cornea Status
1	0.3	Phacocanaloplasty	Non-HDMD	Infero-nasal and infero-temporal	0.5	Postoperative drainage	0.9	Clear
2	0.4	Phacocanaloplasty	HDMD	Infero-temporal	0.7	Observation	1	Remnants of HDMD
3	0.6	Phacocanaloplasty	Non-HDMD	Infero-temporal	0.6	Intraoperative surgical drainage and air injection	0.9	Disruption of DM with no edema

## Data Availability

Data is unavailable due to privacy.
